# A hospital-based study on etiology and prognosis of bacterial meningitis in adults

**DOI:** 10.1038/s41598-021-85382-4

**Published:** 2021-03-16

**Authors:** Jun-Sang Sunwoo, Hye-Rim Shin, Han Sang Lee, Jangsup Moon, Soon-Tae Lee, Keun-Hwa Jung, Kyung-Il Park, Ki-Young Jung, Manho Kim, Sang Kun Lee, Kon Chu

**Affiliations:** 1 Department of Neurosurgery, Seoul National University Hospital, Seoul, South Korea; 2 Department of Neurology, Dankook University Hospital, Cheonan, South Korea; 3 Department of Neurology, Comprehensive Epilepsy Center, Biomedical Research Institute, Seoul National University Hospital, 101, Daehak-ro, Jongno-gu, Seoul, 03080 South Korea; 4 Rare Disease Center, Seoul National University Hospital, Seoul, South Korea; 5 Department of Neurology, Seoul National University Hospital Healthcare System Gangnam Center, Seoul, South Korea; 6 Protein Metabolism and Neuroscience Dementia Medical Research Center, Seoul National University College of Medicine, Seoul, South Korea

**Keywords:** Central nervous system infections, Meningitis

## Abstract

Bacterial meningitis is a neurological emergency with high morbidity and mortality. We herein investigated clinical features, etiology, antimicrobial susceptibility profiles, and prognosis of bacterial meningitis in adults from a single tertiary center. We retrospectively reviewed medical records of patients with laboratory-confirmed bacterial meningitis from 2007 to 2016. Patients with recent neurosurgery, head trauma, or indwelling neurosurgical devices were classified as having healthcare-related meningitis. Causative microorganisms were identified by analyzing cerebrospinal fluid (CSF) and blood cultures, and antimicrobial susceptibility profiles were evaluated. We performed multiple logistic regression analysis to identify factors associated with unfavorable outcomes. We identified 161 cases (age, 55.9 ± 15.5 years; male, 50.9%), of which 43 had community-acquired and 118 had healthcare-related meningitis. CSF and blood culture positivity rates were 91.3% and 30.4%, respectively. In community-acquired meningitis patients, *Klebsiella pneumoniae* (25.6%) was the most common isolate, followed by *Streptococcus pneumoniae* (18.6%) and *Listeria monocytogenes* (11.6%). The susceptibility rates of *K. pneumoniae* to ceftriaxone, cefepime, and meropenem were 85.7%, 81.3%, and 100%, respectively. Among healthcare-related meningitis patients, the most common bacterial isolates were coagulase-negative staphylococci (28.0%), followed by *Staphylococcus aureus* (16.1%) and *Enterobacter* spp. (13.6%). Neurological complications occurred in 39.1% of the patients and the 3-month mortality rate was 14.8%. After adjusting for covariates, unfavorable outcome was significantly associated with old age (odds ratio [OR] 1.03, 95% confidence interval [CI] 1.00–1.06), neurological complications (OR 4.53, 95% CI 1.57–13.05), and initial Glasgow coma scale ≤ 8 (OR 19.71, 95% CI 4.35–89.40). Understanding bacterial pathogens and their antibiotic susceptibility may help optimize antimicrobial therapy in adult bacterial meningitis.

## Introduction

Bacterial meningitis is a neurological emergency with high morbidity and mortality. Over 1.2 million cases of bacterial meningitis are estimated to occur annually worldwide^1^. Although adjunctive dexamethasone reduces the risk of unfavorable outcomes and death, neurological complications occur in approximately 30% of survivors^2,3^. Delayed antibiotic administration has been shown to significantly increase mortality and adverse outcomes at 3 months^4^. Therefore, early clinical suspicion and immediate antibiotic therapy are crucial in the initial management of bacterial meningitis.

Antibiotic treatments are determined empirically, based on the common causative pathogens of bacterial meningitis, age, host immune status, and predisposing conditions^5^. According to previous epidemiological studies, *Streptococcus pneumoniae*, *Neisseria meningitidis*, *Haemophilus influenzae*, and *Listeria monocytogenes* are the major bacterial pathogens responsible for community-acquired meningitis in adults^6,7^. On the other hand, the most common microorganisms associated with neurosurgical procedures and head trauma were coagulase-negative staphylococci (CoNS), *Staphylococcus aureus*, and gram-negative bacilli^8^. However, the epidemiology of bacterial meningitis has changed over the past 30 years. The introduction of conjugate vaccines significantly decreased the incidence of *H. influenzae* and *S. pneumoniae* meningitis and also shifted the age distribution of bacterial meningitis from children to older adults^9,10^. The increasing rate of antimicrobial resistance in *S. pneumoniae* is another important epidemiological trend that should be considered when selecting the appropriate antibiotic therapy^11^. However, there is little information on the etiology and antimicrobial susceptibility profiles of recent bacterial meningitis cases, especially in Korea. Therefore, we investigated the clinical, laboratory, and microbiological profiles of adult bacterial meningitis patients from a single tertiary center over a 10-year period.

## Methods

### Study subjects

We retrospectively reviewed the medical records of adult patients with laboratory-confirmed bacterial meningitis, who were treated in Seoul National University Hospital from 2007 to 2016. Bacterial meningitis was defined as follows according to the World Health Organization recommendation^12^. Suspected cases were defined as any person with clinical features of bacterial meningitis, such as fever, altered consciousness, and meningeal signs. Probable cases were defined as any suspected cases with cerebrospinal fluid (CSF) white blood cell (WBC) count > 100 cells/mm^3^, or CSF WBC count of 10–100 cells/mm^3^ with either protein level > 100 mg/dL or glucose level < 40 mg/dL. Finally, laboratory-confirmed cases were defined as any suspected or probable cases in which bacterial pathogens were identified in CSF or blood cultures or bacterial antigen detection by CSF latex agglutination test. Only laboratory-confirmed bacterial meningitis cases were selected as study subjects and included in the analysis. Patients with tuberculous meningitis and meningoencephalitis due to non-bacterial pathogens were excluded from the study. When CSF and blood cultures showed discordant results, the isolate from the CSF culture was considered as the causative organism.

Bacterial meningitis cases were classified into community-acquired and healthcare-related meningitis, because they have a different spectrum of bacterial pathogens. Patients with recent neurosurgery (within 1 month of the onset of meningitis); head trauma; or indwelling neurosurgical devices, such as ventriculoperitoneal shunt, extraventricular drain, and lumbar drain, were classified as having healthcare-related meningitis. Patients without evidence of healthcare-related infection were classified as having community-acquired meningitis.

### Clinical information

We analyzed demographic information, symptoms and signs at presentation, premorbid functional status, immunocompromised status, concurrent infection, indwelling neurosurgical devices, and recent neurosurgery or head trauma. Severe mental deterioration at admission was defined as an initial Glasgow Coma Scale (GCS) score ≤ 8. We evaluated the initial CSF profiles, including cell count with differential and protein and glucose levels. Clinical outcomes were measured using a modified Rankin Scale (mRS) score at discharge and 3 months after discharge. An unfavorable outcome was defined as an mRS score ≥ 4 at 3 months.

### Antimicrobial susceptibility test

Antimicrobial susceptibility tests were performed and interpreted according to the Clinical and Laboratory Standards Institute. The results were reported as susceptible, intermediate, or resistant. A bacterial isolate was classified as non-susceptible to an antimicrobial agent when it tested as intermediate or resistant. Multi-drug resistance (MDR) was defined, according to the guidelines of the European Centre for Disease Prevention and Control^13^, as non-susceptibility to at least one agent in three or more antimicrobial categories. In particular, MDR of *Streptococcus* spp. was defined as non-susceptibility to penicillin and antimicrobials in two or more other non-β-lactam classes^14^.

### Ethical statement

This study protocol was approved by the institutional review board (IRB) of Seoul National University Hospital (No. C-1705–016-851) and was performed in accordance with the principles of the Declaration of Helsinki. Because this was a retrospective medical chart review study, informed consent was not obtained from the participants and the IRB of Seoul National University Hospital granted a waiver of informed consent. All information gathered in this study was anonymized to preserve the participants’ privacy.

### Statistical analysis

We performed a Student's t-test or a Pearson's chi-square test for between-group comparisons of continuous and categorical variables, respectively. Data that were not normally distributed are presented as median (interquartile range) and were analyzed using a Wilcoxon rank-sum test. We performed multivariate logistic regression analyses to identify factors related to clinical outcomes in bacterial meningitis patients. Dependent variables were unfavorable outcome and mortality at 3 months, analyzed separately. Patients with a premorbid disability, defined as a premorbid mRS score ≥ 3, were excluded from the analysis. Variables with *P* < 0.1 in univariate logistic analyses were included as independent variables. In addition, age, sex, and type of meningitis (healthcare-related vs community-acquired) were included as covariates. A two-tailed *P*-value < 0.05 was considered statistically significant and statistical analyses were performed using SPSS version 25 (IBM Corp. Armonk, NY, USA).

## Results

### Clinical presentation

We identified 161 cases of which 43 had community-acquired meningitis and 118 had healthcare-related meningitis. Six patients, all of whom had a healthcare-related infection, experienced a second episode of bacterial meningitis. Of these, postoperative CSF leak occurred in one patient and intraventricular devices were implanted in the other five patients. Overall, the mean age was 55.9 ± 15.5 years and 50.9% of patients were male (Table 1). There was no significant seasonal variation (*P* = 0.806). Regarding predisposing factors, immunocompromised conditions were present in 31 (19.3%) patients and a concurrent infection, such as pneumonia, catheter-related blood stream infection, or peritonitis, was found in 44 (27.3%) patients. Among the healthcare-related meningitis patients, 64.4% underwent recent neurosurgery and 62.7% had indwelling neurosurgical devices. At presentation, the classic triad of fever, neck stiffness, and altered mental status was found in 31.3% of patients. The initial GCS score was 12.3 ± 3.8 and severe mental deterioration was observed in 33 (20.5%) patients. Compared to healthcare-related patients, community-acquired meningitis patients were characterized by older age (*P* < 0.001), lower initial GCS scores (*P* = 0.032), and a higher rate of neck stiffness (*P* = 0.049) and the classic symptom triad (*P* = 0.003). Regarding predisposing conditions, patients with healthcare-related meningitis showed a higher prevalence of concurrent infections (*P* = 0.027) and a lower prevalence of diabetes mellitus (*P* = 0.034) than those with community-acquired meningitis.

**Table 1:** Clinical features of bacterial meningitis at presentation.

Variables	Total (n = 161)	Community-acquired (n = 43)	Healthcare-related (n = 118)	*P*
Age, years	55.9 ± 15.5	64.6 ± 12.5	52.7 ± 15.3	< 0.001
Male	50.9%	60.5%	47.5%	0.144
Body temperature, °C	37.6 ± 1.0	37.6 ± 1.1	37.6 ± 1.0	0.948
Body temperature ≥ 38 °C	38.0%	37.2%	38.3%	0.904
Fever by history	88.2%	93.0%	86.4%	0.252
Neck stiffness*	62.5%	73.7%	52.4%	0.049
Mental status change	52.2%	74.4%	44.1%	0.001
GCS score	12.3 ± 3.8	11.2 ± 4.0	12.7 ± 3.7	0.032
GCS score ≤ 8	20.5%	30.2%	16.9%	0.065
Symptom triad*	31.3%	47.4%	16.7%	0.003
Systolic BP, mmHg	134.5 ± 26.1	142.4 ± 29.8	131.6 ± 24.1	0.037
Diastolic BP, mmHg	79.4 ± 16.0	81.3 ± 13.9	78.7 ± 16.7	0.373
**Predisposing factors**				
Immunocompromised	19.3%	11.6%	22.0%	0.138
Concurrent infections	27.3%	14.0%	32.2%	0.027
Diabetes mellitus	17.4%	27.9%	13.6%	0.034
Recent neurosurgery†	47.2%	0	64.4%	< 0.001
Neurosurgical devices	46.0%	0	62.7%	< 0.001
CSF leakage	7.5%	2.3%	9.3%	0.184

Data are presented as mean ± SD or %. *Data were available in 85 cases. †Recent neurosurgery refers to neurosurgery within 1 month of the onset of meningitis. Abbreviations: GCS, Glasgow coma scale; CSF, cerebrospinal fluid.

### Laboratory findings

Mean CSF opening pressure was 22.5 ± 10.6 cmH_2_O, and an elevated pressure ≥ 20 cmH_2_O was found in 54.3% of the patients (Table 2). Median CSF WBC count was 828.0/mm^3^ (interquartile range [IQR], 256.3–2870.0), with 76.3% neutrophils and 14.0% lymphocytes. The median protein level was 201.8 mg/dL (IQR, 93.0–489.0) and CSF/blood glucose ratio was 0.28 (IQR, 0.07–0.47). Community-acquired meningitis patients showed higher CSF protein levels (*P* = 0.005) and lower CSF glucose levels (*P* = 0.002) than healthcare-related meningitis patients, although CSF WBC counts did not significantly differ between the two groups. In blood tests, thrombocytopenia, with a platelet count < 100, 000/mm^3^, was noted in 12.6% of the patients, whereas increased high-sensitivity C-reactive protein (hs-CRP) levels > 10 mg/dL were identified in 42.8% of the patients.

**Table 2:** Cerebrospinal fluid and blood test findings.

Variables	Total (n = 161)	Community-acquired (n = 43)	Healthcare-related (n = 118)	*P*
**CSF profiles**				
Pressure, cmH_2_O	22.5 ± 10.6	22.9 ± 8.9	22.1 ± 12.3	0.753
WBC count, / mm^3^	828.0 (256.3–2870.0)	1215.0 (234.0–4464.0)	800.0 (257.0–2690.0)	0.394
≤ 99/ mm^3^	12.5%	11.6%	12.8%	
100–999/ mm^3^	78.8%	76.7%	79.5%	
≥ 1000/ mm^3^	8.8%	11.6%	7.7%	
Neutrophils, %†	76.3 ± 26.1	80.6 ± 26.0	74.8 ± 26.1	0.222
Protein, mg/dL	201.8 (93.0–489.0)	332.0 (165.0–523.0)	177.0 (82.8–405.9)	0.005
Glucose, mg/dL	44.0 (10.0–68.0)	21.0 (5.0–52.0)	51.0 (20.0–69.3)	0.002
Glucose ratio (CSF/blood), %*	27.8 (6.8–47.3)	14.9 (4.0–28.7)	34.2 (11.6–51.2)	< 0.001
**Blood tests†**				
Platelet count, × 10^3^/mm^3^	218.7 ± 104.1	159.9 ± 92.3	240.5 ± 100.0	< 0.001
hs-CRP, mg/dL	10.8 ± 10.9	15.3 ± 13.7	9.1 ± 9.1	0.009

Data are presented as mean ± SD, median (interquartile range), or %. *Data were available in 156cases. †Data were available in 159cases. Abbreviations: CSF, cerebrospinal fluid; WBC, white blood cell; hs-CRP, high-sensitivity C-reactive protein.

### Causative microorganisms

Overall, CSF Gram stains and cultures were positive in 24.5% (34/139) and 91.3% (147/161) of patients, respectively. Blood cultures were positive in 30.4% (49/161) of patients and blood and CSF cultures were both positive in 21.7% (35/161) of patients. Bacterial antigen detection tests were only performed in 40 patients and six of these (15.0%) were positive, with five testing positive for *S. pneumoniae* and one testing positive for *Streptococcus agalactiae*.

The bacterial pathogens isolated from 161 bacterial meningitis cases are summarized in Table 3. Mixed infections with two different species were found in four patients. In community-acquired meningitis patients, *Klebsiella pneumoniae* (25.6%) was the most common isolate, followed by *S. pneumoniae* (18.6%) and *L. monocytogenes* (11.6%). *H. influenzae* accounted for 4.7% of infections, but *N. meningitidis* was not detected. Among healthcare-related meningitis patients, the most common bacterial isolates were CoNS (28.0%), followed by *S. aureus* (16.1%) and *Enterobacter* spp. (13.6%). *Streptococcus* spp. were more common in community-acquired meningitis patients (34.9% vs. 4.2%, *P* < 0.001), whereas *Staphylococcus* spp. were more frequently isolated from healthcare-related meningitis patients (44.1% vs. 4.7%, *P* < 0.001). Furthermore, *L. monocytogenes* was only isolated from community-acquired meningitis patients, whereas CoNS and *Enterobacter* spp. were only isolated from healthcare-related meningitis patients.

**Table 3:** Causative microorganisms.

Microorganisms	Community-acquired (n = 43)	Healthcare-related (n = 118)	*P*
**Gram positive**			
*Streptococcus* spp.	34.9%	4.2%	< 0.001
*S. pneumoniae*	18.6%	1.7%	
Viridans streptococci	7.0%	2.5%	
* S. agalactiae*	9.3%	0	
*Staphylococcus* spp.	4.7%	44.1%	< 0.001
* S. aureus*	4.7%	16.1%	
* S. epidermidis*	0	17.8%	
Other CoNS	0	10.2%	
Other gram positive	25.6%	11.9%	0.033
* Listeria monocytogenes*	11.6%	0	
* Enterococcus* spp.*	2.3%	6.8%	
* Bacillus* spp.	4.7%	5.1%	
Others†	7.0%	0	
**Gram negative**	34.9%	43.2%	0.341
* Klebsiella pneumoniae*	25.6%	5.9%	
* Pseudomonas* spp.‡	7.0%	6.8%	
* Acinetobacter* spp.∥	2.3%	6.8%	
* Enterobacter* spp.¶	0	13.6%	
Others**	0	10.2%	

Abbreviations: CoNS, Coagulase-negative staphylococci. *E. faecalis (n = 5) and *E. faecium* (n = 4). †*Corynebacterium* species, *Micrococcus* species, and *Parvimonas micra* (n = 1 for all). ‡*P. aeruginosa* (n = 10) and *P. fluorescens* (n = 1). ∥*A. baumannii* (n = 7) and *A. lwoffii* (n = 2). ¶*E. aerogenes* (n = 13) and *E. cloacae* (n = 3). **Serratia marcescenes (n = 4), *Stenotrophomonas maltophilia* (n = 3), *Haemophilus influenzae* (n = 2), *Escherichia coli* (n = 1), *Morganella morganii* (n = 1), and *Moraxella species* (n = 1).

### Antimicrobial susceptibility profiles

The susceptibility rates of *S. pneumoniae* to penicillin G, cefotaxime, and vancomycin were 33.3%, 40.0%, and 100%, respectively (Supplementary Table S1). For streptococci other than *S. pneumoniae*, the susceptibility rates to penicillin and vancomycin were 62.5% and 100%, respectively. Among the *S. aureus* isolates, 85% were resistant to methicillin (oxacillin), but 100% were susceptible to vancomycin. *S. epidermidis* isolates showed similar profiles, with 90.5% resistant to methicillin (oxacillin), but 100% susceptible to vancomycin. The MDR rate of *Staphylococcus* spp. was 88.5%. Among gram-negative bacilli, the susceptibility rates of *K. pneumoniae* to ceftriaxone, cefepime, and meropenem were 85.7%, 81.3%, and 100%, respectively (Supplementary Table S2). Extended-spectrum β-lactamase (ESBL) producers were found to account for 18.8% (3/16) of the *K. pneumoniae* isolates, all of which were from healthcare-related meningitis patients. The MDR rates of *K. pneumoniae*, *Pseudomonas* spp., *Acinetobacter* spp., and *Enterobacter* spp. were 25.0%, 18.2%, 44.4%, and 56.3%, respectively. Overall, bacterial isolates from healthcare-related meningitis patients showed higher rates of MDR than those from community-acquired meningitis patients (69.1% vs. 25.0%, *P* < 0.001).

### Complications and outcomes

The mean length of stay in the hospital was 76.7 ± 97.6 days, with 52.8% of patients treated in the intensive care unit. Mechanical ventilation was used in 36.0% of patients. Neurological complications occurred in 39.1% of patients and the most common complication was hydrocephalus (19.9%), followed by seizures (13.7%). Ischemic infarction and cerebral hemorrhage were more common in community-acquired patients than in healthcare-related patients (*P* < 0.001 and 0.015, respectively; Table 4). Mortality rates at discharge and 3 months after discharge were 10.6% and 14.8%, respectively. Mortality rates and mRS scores at discharge and 3 months after discharge did not differ between community-acquired and healthcare-related meningitis patients, although 3-month mRS scores in healthcare-related meningitis patients tended to be higher than those in community-acquired meningitis patients (*P* = 0.075).

**Table 4:** Complications and outcome of bacterial meningitis cases.

	Total (n = 161)	Community-acquired (n = 43)	Healthcare-related (n = 118)	*P*
**Complications**	39.1%	65.1%	29.7%	< 0.001
Hydrocephalus	19.9%	16.3%	21.2%	0.490
Seizure	13.7%	18.6%	11.9%	0.271
Brain abscess	8.7%	14.0%	6.8%	0.204
Ischemic infarction	5.6%	18.6%	0.8%	< 0.001
Cerebral hemorrhage	4.3%	11.6%	1.7%	0.015
**Outcomes**				
Length of stay, days	76.7 ± 97.6	44.0 ± 36.6	88.6 ± 109.6	< 0.001
ICU admission	52.8%	51.2%	53.4%	0.802
mRS at discharge	3.3 ± 1.9	3.1 ± 1.9	3.4 ± 1.9	0.289
mRS 0–3 at discharge	53.4%	60.5%	50.8%	0.279
In-hospital mortality	10.6%	11.6%	10.2%	0.777
mRS at 3 months*	3.0 ± 2.1	2.5 ± 2.2	3.2 ± 2.0	0.075
mRS 0–3 at 3 months	60.6%	68.4%	57.7%	0.247
Mortality at 3 months	14.8%	18.4%	13.5%	0.461

Data are presented as mean ± SD or %. *3-month outcomes were available in 142 cases. Abbreviations: ICU, intensive care unit; mRS, modified Rankin Scale.

We then assessed factors associated with unfavorable outcomes and mortality at 3 months in adult bacterial meningitis patients. Since 19 patients had no follow-up data at 3 months after discharge, we analyzed the outcome in 142 patients. In univariate analysis, an unfavorable outcome was associated with older age, neurological complications, concurrent infection, high hs-CRP levels, and an initial GCS score ≤ 8. However, neither positive Gram staining results, the MDR status of isolates, nor immunocompromised status were associated with an unfavorable outcome (Supplementary Table S3). After adjusting for covariates, an unfavorable outcome was significantly associated with older age (odds ratio [OR] 1.03, 95% confidence interval [CI] 1.00–1.06), neurological complications (OR 4.53, 95% CI 1.57–13.05), and an initial GCS score ≤ 8 (OR 19.71, 95% CI 4.35–89.40; Table 5). Multivariate analysis for mortality at 3 months showed similar results with higher mortality rates associated with neurological complications (OR 5.67, 95% CI 1.76–18.25) and an initial GCS score ≤ 8 (OR 5.31, 95% CI 1.47–19.11).

**Table 5:** Multivariate analysis for unfavorable outcome and mortality at 3 months.

Variables	Unfavorable outcome	Mortality
	OR (95% CI)*	OR (95% CI)†
Age, years	1.03 (1.00–1.06)	1.02 (0.98–1.06)
Neurological complications	4.53 (1.57–13.05)	5.67 (1.76–18.25)
Initial GCS score ≤ 8	19.71 (4.35–89.40)	5.31 (1.47–19.11)

Patients with premorbid mRS scores 0–2 were analyzed (n = 124). *Sex, healthcare-related meningitis, concurrent infection, and high-sensitivity C-reactive protein > 10 mg/dL were included as covariates. †Sex, healthcare-related meningitis, immunocompromised status, thrombocytopenia < 100,000/mm^3^, and high-sensitivity C-reactive protein > 10 mg/dL were included as covariates. Abbreviations: OR, odds ratio; CI, confidence interval; GCS, Glasgow coma scale.

We additionally performed subgroup analysis on the unfavorable outcome for community-acquired and healthcare-related meningitis, respectively. In healthcare-related meningitis, older age (OR 1.07, 95% CI 1.02–1.11), neurological complications (OR 4.13, 95% CI 1.12–15.25), and an initial GCS score ≤ 8 (OR 39.93, 95% CI 2.61–610.86) were associated with the unfavorable outcome, which is similar to the results from the total subjects. By contrast, an initial GCS score ≤ 8 (OR 14.23, 95% CI 1.82–111.31) only remained significantly associated with the unfavorable outcome in community-acquired meningitis (Table S4).

## Discussion

In this study, we investigated clinical, laboratory, and microbiological profiles of adult bacterial meningitis. The composition of causative microorganisms was significantly different between community-acquired and healthcare-related meningitis. *Streptococcus* spp. accounted for 34.9% of community-acquired meningitis patients, whereas *Staphylococcus* spp. accounted for 44.1% of healthcare-related meningitis patients. At the species level, *K. pneumoniae* (25.6%) was the most common causative bacterium in community-acquired meningitis, followed by *S. pneumoniae* (18.6%) and *L. monocytogenes* (11.6%)*.* In healthcare-related meningitis patients, *S. epidermidis* (17.8%) was the most common causative bacterium, followed by *S. aureus* (16.1%). Mortality during hospitalization and 3 months after discharge were 10.6% and 14.8%, respectively. Older age, any neurological complications, and severe mental deterioration at admission were significantly associated with unfavorable outcomes, which is consistent with the results of previous studies^3,15^.

A notable finding in this study was that *K. pneumoniae* was the most common pathogen in community-acquired meningitis patients. This is consistent with data from Taiwan showing that *K. pneumoniae* was the leading causative pathogen, accounting for 44.9% of spontaneous bacterial meningitis patients^16^. Although a high incidence of *K. pneumoniae* meningitis, together with a low incidence of *S. pneumoniae* meningitis has been reported in Taiwan in the 1980s and 1990s, the exact cause has not been determined^17^. Diabetes mellitus has been reported as a risk factor for community-acquired meningitis and liver abscesses caused by *K. pneumoniae*^18^*.* In agreement with this, 54.5% (6/11) of community-acquired *K. pneumoniae* meningitis patients in our study had comorbid diabetes mellitus. However, this cannot explain the sudden change in the epidemiology of bacterial meningitis in Korea. An epidemiological study in Korean adults between 1998 and 2008 showed that *K. pneumoniae* was the third most common pathogen, but accounted for only 7.7% of community-acquired meningitis cases^19^. Selection bias due to a single-center study design may have contributed to our results. Nevertheless, our findings suggested that *K. pneumoniae* infection should be considered as a possible etiology of community-acquired bacterial meningitis, at least in the tertiary hospital setting. It also cannot be concluded whether the increase in *K. pneumoniae* meningitis is due to regional or racial influences in East Asian countries. Further epidemiological studies in other countries are needed to address this issue. All *K. pneumoniae* isolates from community-acquired meningitis patients were susceptible to cefotaxime and cefepime. ESBL-producing *K. pneumoniae*, all of which were isolated from healthcare-related meningitis patients, were resistant to ceftazidime and cefepime, but susceptible to imipenem and meropenem. These antimicrobial susceptibility profiles are consistent with previous studies^20^, suggesting that the third- and fourth-generation cephalosporins are appropriate for antibiotic therapy against community-acquired meningitis associated with *K. pneumoniae*, but carbapenems should be considered for the treatment of healthcare-related meningitis.

*S. pneumoniae* has traditionally been reported as the most common causative organism of community-acquired bacterial meningitis in adults^3,21^. However, *S. pneumoniae* only accounted for 18.6% of cases in our study, although it was the second most common species detected. The introduction of conjugate vaccines against *S. pneumoniae* is a possible explanation for the low frequency of pneumococcal meningitis. Pneumococcal vaccination significantly decreased the incidence of pneumococcal meningitis in children and adults, suggesting an indirect effect of vaccination through herd immunity^22,23^. The high rate of resistance of *S. pneumoniae* to penicillin and third-generation cephalosporins confirmed that antibiotics containing vancomycin are the appropriate empirical regimen for *S. pneumoniae* meningitis. The low frequency of *H. influenzae* meningitis in this study is also thought to be the result of *H. influenzae* type b vaccination^6^. In Korea, the incidence of *H. influenzae* meningitis in children has significantly decreased since 2001^24^, and in a previous study, *H. influenzae* was not isolated among 196 adult patients with community-acquired meningitis^19^.

*L. monocytogenes* was the third most common pathogen detected in our study population. It is well known that elderly individuals and immunocompromised patients are at a high risk of contracting *L. monocytogenes* meningitis^25^. In our study, the mean age of *L. monocytogenes* meningitis patients was 73.6 years (range, 66–83 years) and two (40%) patients were immunocompromised. The two (40%) immunocompromised patients died in hospital and the other two (40%) were severely disabled (mRS score, 5) at 3 months, thus confirming the high mortality and morbidity rates previously reported for *L. monocytogenes* meningitis^26^.

The frequency of healthcare-related meningitis (73.3%) was higher than that of community-acquired meningitis (26.7%) in our study. These findings are likely to be caused by selecting bacterial meningitis cases from the tertiary hospital. Patients with community-acquired meningitis, especially those with mild severity, might have been treated at a lower level hospital rather than being transferred to this hospital. It is also possible that the epidemiological trend of decreasing frequencies of pneumococcal and meningococcal meningitis contributed to a decrease in the overall number of community-acquired meningitis and a relative increase in the proportion of healthcare-related meningitis^10^. However, it cannot be concluded in this study, and a nationwide epidemiological investigation is required to address this issue. The most common microorganisms causing healthcare-related bacterial meningitis were CoNS, *S. aureus*, and gram-negative bacilli, which were consistent with the results of previous studies^17,21^. *Staphylococcus* spp. were mostly methicillin-resistant, but all were susceptible to vancomycin. Gram-negative bacilli showed moderate susceptibility to third- and fourth-generation cephalosporins, but high susceptibility to carbapenems. These antimicrobial susceptibility profiles support the use of vancomycin plus meropenem for the empirical treatment of healthcare-related meningitis^27^. Exceptionally, *Acinetobacter* spp. showed high resistance (44.4%) to meropenem. Therefore, if the *Acinetobacter* isolates are resistant to carbapenems, colistin or polymyxin B should be administered^28^.

There are several limitations of this study. Firstly, as a single-center study, our subjects may not represent the general population of adult bacterial meningitis patients in Korea. Recruitment from a tertiary hospital may have caused selection bias toward more severe cases. Moreover, although we collected data over a 10-year period, the sample size was relatively small, especially for community-acquired meningitis. Another limitation is the retrospective chart review design, which may have resulted in incomplete or inaccurate data collection. We first investigated the results of CSF examinations and cultures, and among probable cases and culture-positive cases, we reviewed the electrical medical records and selected those who met the definition of laboratory-confirmed bacterial meningitis. Nevertheless, we might have missed some cases, which is an inevitable limitation of the retrospective study. Furthermore, although we identified the clinical factors associated with unfavorable outcomes in bacterial meningitis patients, a causal relationship cannot be inferred from this study. Although we adjusted for the effect of the type of infection in the multivariate analysis, we cannot completely rule out the effect of neurosurgery itself on the unfavorable outcome.

In conclusion, we identified a spectrum of causative microorganisms for adult bacterial meningitis cases over a recent 10-year period. The increased proportion of *K. pneumoniae* infections and the decreased proportion of *S. pneumoniae* infections among community-acquired meningitis patients was particularly noteworthy. Our data on causative microorganisms and their antibiotic susceptibility profiles may help optimize determination of the appropriate empirical antimicrobial therapy for adult bacterial meningitis patients. However, further studies are required to confirm the changing epidemiology of causative pathogens and prognostic factors in adult bacterial meningitis.

## Supplementary Information

Supplementary Information

## Data Availability

Deidentified data are available from the corresponding author upon reasonable request.
