# Supplementary material for: A hospital-based study on etiology and prognosis of bacterial meningitis in adults
Source: Sci Rep. 2021 Mar 16;11:6028. doi: 10.1038/s41598-021-85382-4 (PMC7966379; doi:10.1038/s41598-021-85382-4)
Supplement: Supplementary file 1 — Supplementary Information [file 41598_2021_85382_MOESM1_ESM.docx]

**A Hospital-Based Study on Etiology and Prognosis of Bacterial Meningitis in Adults**

Jun-Sang Sunwoo^1^, Hye-Rim Shin^2^, Han Sang Lee^3^, Jangsup Moon^3,4^, Soon-Tae Lee^3^, Keun-Hwa Jung^3^, Kyung-Il Park^5^, Ki-Young Jung^3^, Manho Kim^3,6^, Sang Kun Lee^3^, Kon Chu^3^

*^1^Department of Neurosurgery, Seoul National University Hospital, Seoul, South Korea*

*2Department of Neurology, Dankook University Hospital, Cheonan, South Korea*

*^3^Department of Neurology, Comprehensive Epilepsy Center, Biomedical Research Institute, Seoul National University Hospital, Seoul, South Korea*

*^4^Rare Disease Center, Seoul National University Hospital, Seoul, South Korea*

*^5^Department of Neurology, Seoul National University Hospital Healthcare System Gangnam Center, Seoul, South Korea*

*^6^Protein Metabolism and Neuroscience Dementia Medical Research Center, Seoul National University College of Medicine, Seoul, South Korea*

**Supplementary Materials**

**Table S1**. Antibiotic susceptibility rate of gram-positive bacterial isolates

**Table S2**. Antibiotic susceptibility rate of gram-negative bacterial isolates

**Table S3**. Univariate analysis for unfavorable outcome and mortality at 3 months

**Table S4**. Subgroup analysis for unfavorable outcome at 3 months

**Table S1**. Antibiotic susceptibility rate of gram-positive bacterial isolates

| Antibiotics | *S. pneumoniae* (n = 9) | Viridans streptococci  (n = 5) | *S. agalactiae*  (n = 3) | *S. aureus*  (n = 20) | *S. epidermidis*  (n = 21) | Other CoNS*  (n = 11) |
| --- | --- | --- | --- | --- | --- | --- |
| Penicillin G | 33.3 | 40.0 | 100 | 5 | 0 | 9.1 |
| Oxacillin | N/A | N/A | N/A | 15 | 9.5 | 27.3 |
| Amoxicillin/Clavulanic acid | 25.0 | N/A | N/A | 15 | 10 | 22.2 |
| Cefuroxime | 0 | N/A | N/A | 12.5 | 0 | 28.6 |
| Cefotaxime | 40.0 | N/A | 100 | 12.5 | 0 | 28.6 |
| Cefepime | 25.0 | N/A | 100 | 12.5 | 0 | 28.6 |
| Erythromycin | 11.1 | 60.0 | 100 | 45 | 10 | 54.5 |
| Clindamycin | 0 | 60.0 | 66.6 | 45 | 52.4 | 36.4 |
| Meropenem | 25.0 | N/A | 100 | 12.5 | 0 | 28.6 |
| Gentamicin | N/A | N/A | N/A | 40 | 23.8 | 27.3 |
| Rifampin | N/A | N/A | N/A | 95 | 100 | 90.9 |
| Chloramphenicol | 75.0 | 80.0 | 100 | 62.5 | 71.4 | 100 |
| Levofloxacin | 100 | N/A | 100 | 45 | 55 | 54.5 |
| Tetracycline | 25.0 | N/A | 100 | 50 | 45 | 45.5 |
| Trimethoprim/Sulfamethoxazole | 33.3 | N/A | 100 | 100 | 85.7 | 18.2 |
| Vancomycin | 100 | 100 | 100 | 100 | 100 | 100 |
| Teicoplanin | N/A | N/A | N/A | 100 | 85.7 | 100 |
| Linezolid | N/A | N/A | N/A | 100 | 100 | 100 |
| Multi-drug resistance | 44.4 | 40.0 | 0 | 85.0 | 95.2 | 81.8 |

Number of antibiotic-susceptible isolates/number tested (%). Abbreviations: N/A, not available; CoNS, coagulase-negative staphylococci. *CoNS other than *S. epidermidis*.

**Table S2**. Antibiotic susceptibility rate of gram-negative bacterial isolates

| Antibiotics | *K. pneumoniae*  (n = 16) | *Pseudomonas* spp. (n = 11) | *Acinetobacter* spp. (n = 9) | *Enterobacter* spp.  (n = 16) |
| --- | --- | --- | --- | --- |
| Ampicillin | 0 | N/A | N/A | 0 |
| Piperacillin | 11.1 | 77.8 | 57.1 | 55.6 |
| Ampicillin/Sulbactam | 71.4 | N/A | 66.7 | 16.7 |
| Piperacillin/Tazobactam | 87.5 | 88.9 | 57.1 | 75.0 |
| Ticarcillin/Clavulanic acid | 71.4 | N/A | 50.0 | 66.7 |
| Cefazolin | 78.6 | N/A | N/A | 0 |
| Cefuroxime | 71.4 | N/A | N/A | 50.0 |
| Cefotaxime | 81.3 | 16.7 | 66.7 | 68.8 |
| Ceftazidime | 75.0 | 63.6 | 55.6 | 75.0 |
| Ceftriaxone | 85.7 | 0 | 66.7 | 83.3 |
| Cefepime | 81.3 | 63.6 | 44.4 | 100 |
| Cefotetan | 100 | N/A | N/A | 66.7 |
| Aztreonam | 85.7 | 44.4 | N/A | N/A |
| Imipenem | 100 | 90.9 | 55.6 | 100 |
| Meropenem | 100 | 90.9 | 55.6 | 100 |
| Amikacin | 93.8 | 81.8 | 85.7 | 100 |
| Gentamicin | 87.5 | 66.7 | 55.6 | 93.8 |
| Tobramycin | 71.4 | 80.0 | 55.6 | N/A |
| Ciprofloxacin | 87.5 | 80.0 | 50.0 | 100 |
| Levofloxacin | 71.4 | 66.7 | 66.7 | 100 |
| Trimethoprim/Sulfamethoxazole | 93.8 | N/A | 66.7 | 100 |
| Tetracycline | 85.7 | N/A | N/A | 100 |
| Colistin | N/A | N/A | 100 | 100 |
| Multi-drug resistance | 25.0 | 18.2 | 44.4 | 56.3 |

Number of antibiotic-susceptible isolates/number tested (%). Abbreviations: N/A, not available.

**Table S3**. Univariate analysis for unfavorable outcome and mortality at 3 months

| Variables | Unfavorable outcome | Mortality |
| --- | --- | --- |
|  | crude OR (95% CI) | crude OR (95% CI) |
| Age, years | 1.03 (1.00-1.06)* | 1.02 (0.99-1.05) |
| Sex, male | 1.10 (0.52-2.29) | 1.28 (0.50-3.30) |
| Immunocompromised status | 1.18 (0.47-2.98) | 2.56 (0.90-7.28) |
| Diabetes mellitus | 0.62 (0.23-1.71) | 0.42 (0.09 - 1.93) |
| Healthcare-related meningitis | 1.68 (0.74-3.81) | 0.76 (0.29-2.00) |
| Concurrent infection | 2.39 (1.03-5.55)* | 1.33 (0.47-3.81) |
| Positive gram stain | 1.60 (0.62-4.15) | 0.74 (0.19-2.85) |
| Multi-drug resistance | 1.48 (0.66-3.29) | 1.20 (0.44-3.30) |
| Neurological complications | 2.39 (1.12-5.11)* | 4.30 (1.59-11.66)** |
| Initial GCS score ≤ 8 | 8.25 (2.74-24.80)*** | 4.72 (1.62-13.71)** |
| Thrombocytopenia (<100K/mm^3^) | 1.46 (0.54-3.94) | 2.77 (0.91-8.41) |
| hs-CRP > 10 mg/dL | 3.24 (1.50-6.98)** | 2.67 (1.01-7.01)* |
| CSF WBC count, /mm^3^ | 1.00 (1.00-1.00) | 1.00 (1.00-1.00) |
| CSF protein, mg/dL | 1.00 (1.00-1.00) | 1.00 (1.00-1.00) |
| CSF glucose, mg/dL | 0.99 (0.99-1.01) | 0.99 (0.97-1.00) |
| Glucose ratio (CSF/blood) | 0.99 (0.98-1.01) | 0.97 (0.95-1.00) |

*P < 0.05, **P < 0.01, ***P < 0.001. Abbreviations: OR, odds ratio; CI, confidence interval; GCS, Glasgow coma scale; hs-CRP, high-sensitivity C-reactive protein; CSF, cerebrospinal fluid; WBC, white blood cell.

**Table S4**. Subgroup analysis for unfavorable outcome at 3 months

| Variables | Community-acquired meningitis (n = 41) | Healthcare-related meningitis (n = 83) |
| --- | --- | --- |
| Age, years | 1.07 (0.97-1.18) | 1.07 (1.02-1.11) |
| Neurological complications | 9.53 (0.86-105.63) | 4.13 (1.12-15.25) |
| Initial GCS score ≤ 8 | 14.23 (1.82-111.31) | 39.93 (2.61-610.86) |

Data are presented as odds ratio (95% confidence interval). Sex, healthcare-related meningitis, concurrent infection, and high-sensitivity C-reactive protein > 10 mg/dL were included as covariates. Abbreviations: GCS, Glasgow coma scale.
